# Mechanisms of Immune Checkpoint Inhibitor-Mediated Colitis

**DOI:** 10.3389/fimmu.2021.768957

**Published:** 2021-10-29

**Authors:** Harm Westdorp, Mark W. D. Sweep, Mark A. J. Gorris, Frank Hoentjen, Marye J. Boers-Sonderen, Rachel S. van der Post, Michel M. van den Heuvel, Berber Piet, Annemarie Boleij, Haiko J. Bloemendal, I. Jolanda M. de Vries

**Affiliations:** ^1^ Department of Tumor Immunology, Radboud Institute for Molecular Life Sciences, Radboud University Medical Centre, Nijmegen, Netherlands; ^2^ Department of Medical Oncology, Radboud University Medical Centre, Nijmegen, Netherlands; ^3^ Oncode Institute, Nijmegen, Netherlands; ^4^ Department of Gastroenterology, Radboud University Medical Centre, Nijmegen, Netherlands; ^5^ Division of Gastroenterology, University of Alberta, Edmonton, AB, Canada; ^6^ Department of Pathology, Radboud Institute for Molecular Life Sciences, Radboud University Medical Centre, Nijmegen, Netherlands; ^7^ Department of Pulmonary Diseases, Radboud University Medical Centre, Nijmegen, Netherlands

**Keywords:** immune checkpoint inhibitor (ICI), immune-related adverse events, colitis, mechanisms, treatment

## Abstract

Immune checkpoint inhibitors (ICIs) have provided tremendous clinical benefit in several cancer types. However, systemic activation of the immune system also leads to several immune-related adverse events. Of these, ICI-mediated colitis (IMC) occurs frequently and is the one with the highest absolute fatality. To improve current treatment strategies, it is important to understand the cellular mechanisms that induce this form of colitis. In this review, we discuss important pathways that are altered in IMC in mouse models and in human colon biopsy samples. This reveals a complex interplay between several types of immune cells and the gut microbiome. In addition to a mechanistic understanding, patients at risk should be identifiable before ICI therapy. Here we propose to focus on T-cell subsets that interact with bacteria after inducing epithelial damage. Especially, intestinal resident immune cells are of interest. This may lead to a better understanding of IMC and provides opportunities for prevention and management.

## Introduction

Immune checkpoint inhibitors (ICIs), such as anti-programmed cell death-1 (PD-1), anti-programmed cell death ligand-1 (PD-L1), and anti-cytotoxic T-lymphocyte antigen-4 (CTLA-4), have revolutionized the treatment of cancer in the past decades. ICI therapy resulted in overall survival benefit for patients with advanced stage cancer, shifting standard clinical practice (1). ICIs are now often administered instead of or along with conventional therapies, such as chemotherapy and radiation therapy, in several advanced cancer types (2).

ICIs release the brake of the immune system during priming of naive T-cells [anti-CTLA-4, but more recently also shown for anti-PD-(L)1 (3, 4)] and during reactivation of memory anti-cancer T-cell responses (anti-PD-(L)1), rather than inducing direct tumor cell death as conventional therapies. However, one may argue that ICIs work by normalization rather than enhancement of the immune system (5). This means that an immune defect, in this case inactivation of T-cells, is normalized. Naive T-cell activation needs three signals: I) T-cell receptor binding to an antigen presented in the context of MHC; II) a signal mostly generated by binding of costimulatory molecules CD80 and/or CD86 on antigen presenting cells (APCs) to receptors of the B7 family (6), and III) cytokine-derived signals mediating T-cell differentiation and expansion (7).

ICI antibodies interfere during different time points of T-cell activation. CTLA-4 is a costimulatory molecule that negatively regulates activation of T-cells. It is a direct antagonist of CD28 (8). CTLA-4 is frequently expressed on regulatory T-cells (Tregs) (9). In mouse models the important role of CTLA-4 expression by Tregs is demonstrated: CTLA-4 deficiency leads to fatal auto-immunity (10). Blocking of the CTLA-4 receptor with ipilimumab, a clinically approved monoclonal IgG1 antibody (11), increases the number of CD4^+^ and CD8^+^ T-cells (12). It was debated for a long time whether anti-CTLA-4 therapy causes depletion of Tregs. In a prospective study in humans, the ratio of CD8^+^ T-cell/Treg increased due to anti-CTLA-4 treatment. However, the density of Tregs in the tumor increased upon anti-CTLA-4 treatment in most cancer types studied (13). Increased levels of Tregs are also observed in patients with autosomal dominant immune dysregulation syndrome due to *CTLA4* mutations. The Tregs in these patients were not functional, most likely related to the inability of the CTLA-4 protein to bind and antagonize the T-cell costimulatory molecule CD80. In contrast to healthy controls, Tregs from these patients were not able to inhibit proliferation of CD4^+^ T-cells (14). Although patients with germline *CTLA4* gene variants and response of cancer patients to ICI therapy are fundamentally very different, both result in an impairment of CTLA-4 binding, impacting the function of Tregs.

PD-1 and the known PD-1 ligands, PD-L1 and PD-L2, are immune checkpoint proteins involved in cell-cell interaction and downstream signal transduction. PD-1 expression has been well characterized on T-cells. Upon binding to PD-L1, T-cell proliferation is inhibited or T-cells are inactivated by inducing a state of anergy (15, 16). PD-L1 is expressed on almost all tumors, as well as on T-cells, B-cells, DCs, and macrophages. In some tumor types PD-L1 expression has proven utility as a predictive response biomarker, whereas certain PD-L1 positive patients do not respond to anti-PD-(L)1 therapies (17). Nevertheless, assessment of PD-L1 expression on protein level on tumor tissue has become clinical practice even though its predictive value is moderate at best. Methods to detect and quantify tumor PD-L1 expression vary greatly (18). The expression and function of PD-L2 is rather similar to PD-L1 (19). PD-L2 is mainly expressed on DCs and macrophages (20). Its expression is also observed in several solid tumors and in hematologic malignancies (21). PD-1 is blocked with FDA- and EMA-approved antibodies nivolumab, pembrolizumab, and cemiplimab, and PD-L1 with atezolizumab, avelumab, and durvalumab (22–27). There are no approved drugs that target PD-L2 directly. Blocking the PD-(L)1 axis leads to increased numbers of CD8^+^ cells, predominantly near the tumor site, with high expression of the cytotoxic granzyme B pathway (28).

Taken together, described anti-CTLA-4 and anti-PD-(L)1 antibodies restore the ability of the immune system to attack the tumor. However, this systemic activation of immune cells and induction of potentially self-reactive T-cells also leads to off-target activity.

## Immune-Related Adverse Events (irAEs)

Dual ICI therapy with anti-CTLA-4 and anti-PD-1 antibodies frequently leads to severe irAEs in more than half of the patients (29, 30). All-grade irAEs have been reported in up to 90% of patients receiving both ICIs (30, 31). IrAEs range from mild (50-90%) to severe (10-50%) according to Common Terminology Criteria for Adverse Events (CTCAE). Common immunotoxicity includes dermatitis, rash, endocrinopathy, diarrhea, colitis, hepatitis, and pneumonitis (32, 33). Of these, ICI-mediated colitis (IMC) most frequently requires discontinuation of ICI therapy and is also responsible for at least 3 out of 10 fatal irAEs (33, 34). This particular inflammation in the colon is often characterized by excessive, watery diarrhea, possibly with blood or mucus in the stool, or abdominal pain (35). As discussed, anti-CTLA-4 therapy leads to more naïve T-cell priming, hence expected to be more frequently accompanied with systemic adverse events, such as IMC. Indeed, a higher occurrence of high-grade ICI-mediated diarrhea (IMD) or IMC is observed after ipilimumab monotherapy (15%) compared to anti-PD-1 monotherapy (3%) in patients with metastatic melanoma and non-small cell lung cancer. In combination therapy with anti-CTLA-4 and anti-PD-1 severe IMD/IMC was observed in 17% of treated patients (30).

Ideally, one would like to be able to restore homeostasis in irAE tissues while maintaining an antitumor response, or to be able to predict which patients are at risk of severe irAE development. To do so, understanding the origin and mechanisms of action of irAEs is essential. In this review, we discuss the current knowledge on mechanisms, biomarkers, and risk factors of IMC. Based on our review of the existing literature, we make recommendations for future research aimed at enhancing fundamental knowledge of the mechanisms and risks of IMC development.

## Mechanisms of IMC Development

While the antitumor mechanisms of ICIs have been carefully studied, large studies trying to unravel the mechanisms involved in irAEs are still lacking. The clinical picture of IMC is often considered comparable to inflammatory bowel diseases (IBD), but there are also many differences. Normal colonic mucosa consists of a normocellular inflammatory infiltrate, which is a mixture of lymphocytes, plasma cells, eosinophilic granulocytes, and histiocytes. In IBD there is an increase in cells, predominantly more plasma cells and neutrophilic granulocytes. In patients with IMC, an increase in cell numbers, intraepithelial lymphocytes, and neutrophilic granulocytes is observed (36). For a better understanding of IMC, and to gain insight in possible differences between ICI therapies in IMC, it is imperative to understand the mechanisms by which IMC is developed in these patients.

### Immune Cell Profile

A CTLA-4 deficiency downregulates Treg functionality in mice, leading to resistance to the inhibitory effects of Tregs on CD4^+^ and CD8^+^ T-cell induction (10). Accordingly, an increased frequency of activated CD4^+^ and CD8^+^ T-cells with a concomitant decrease in naive T-cell populations was seen in blood of ipilimumab-treated patients (12, 13). Histopathologic features of IMC patients treated with ipilimumab showed mainly neutrophilic inflammation, but also increased CD4^+^ cells in the lamina propria and increased CD8^+^ cells within the crypt epithelium were observed (36). A recent study by Luoma et al. has shown that in particular the numbers of cytotoxic T-lymphocytes (CTLs) and proliferating T-cells (Ki-67^+^) were increased in IMC biopsies following ipilimumab monotherapy or ICI combination therapy (37). In contrast, tissue-resident memory (Trm) T-cells, a T-cell subset that does not recirculate (38), were reduced in IMC patients as a fraction of total T cells. Interestingly, ICI treated patients who did not develop IMC did not show changes in colonic Trm cells. In IMC patients only, T-cell receptor clonotypes overlapped between CD8^+^ Trm cells and CTLs, suggesting differentiation from the former to the latter (37). This might indicate that there is a shift from CD8^+^ Trm cells towards CTLs in patients with IMC specifically. In non-small cell lung carcinoma, Trm cells have indeed shown to be capable of becoming cytotoxic (39). These potentially Trm-derived CTLs of IMC patients exhibited a genetic profile strongly related to an interferon gamma (IFNγ)-mediated T-helper 1 (Th1) response (37). If IFNγ is indeed abundantly secreted by CTLs in IMC, this could cause disruption of the epithelial barrier function or even apoptosis of human colonic epithelial cells, as shown in *in vitro* models (40, 41). This might explain colonic inflammation and damage that is seen in colonoscopies.

Under normal circumstances, Tregs are able to suppress intestinal inflammation (42), which is evidently compromised in IMC. Similarly to intratumoral Tregs (13), in colonic biopsies of patients with IMC, ipilimumab treatment tends to increase the number of Tregs, defined as FOXP3^+^ cells (43, 44). In a study with IMC patients who received combination therapy, an altered genetic Treg expression profile was seen. These alterations were considered beneficial for suppressing an IFNγ-mediated Th1 response (37). Likewise, elevated mRNA expression of interleukin-10 (IL-10) has been reported in colonic mucosa of IMC patients after anti-CTLA-4 treatment (44). This cytokine is typically secreted by Tregs to dampen inflammation and is an important mediator to suppress colon inflammation (45). However, IL-10 is regulated by various factors on the posttranscriptional level, and its mRNA stability and degradation may vary immensely based on extrinsic signals (46, 47). Thus, while Tregs of IMC patients show expression of Th1-suppressive mechanisms, it may very well be attenuated at the translational or protein level, thereby limiting Treg functionality.

In the context of reduced Treg-mediated immune suppression, Th17 cells may become more pronounced in IMC. Th17 cells are capable of developing colitis in mouse models when the IL-10 receptor (IL-10R) is deleted in Tregs (48), highlighting the importance of IL-10 in maintaining intestinal homeostasis. In addition to IL-10, CTLA-4 is required for Tregs to suppress Th17 cells (48, 49). Inability to suppress Th17 cells possibly explains why CTLA-4 blockade leads to increased mucosal IL-10 mRNA in IMC biopsies without successfully resolving IMC (44). Th17 cells, which are potent secretors of IL-17, are present in IMC. Serum IL-17 levels correlated strongly with ipilimumab-induced IMC, from onset to resolution, while the other examined cytokines did not express such a pattern (50). Parallel to serum levels, in ipilimumab-induced IMC IL-17A mRNA is significantly increased in colonic biopsies, as is similar to IBD (44). Together, these findings indicate an important role for Th17 cells in IMC.

The Th17/IL-17 axis is, amongst others, responsible for production of the chemokines CXCL8 and GM-CSF by intestinal epithelial cells (51). These chemokines attract neutrophils and prevent their apoptosis, employing them as a mucosal barrier defense (52–54). Neutrophil infiltration in the epithelial layers is indeed a characteristic of human IMC biopsies after both anti-CTLA-4 (36) and anti-PD-1 therapy (55). Th17-mediated neutrophil recruitment may thus be an important mechanism of inflammation in IMC. Furthermore, the mouse equivalent of human CXCL1, an important chemokine for neutrophil recruitment (56, 57), was found in serum following ICI therapy in colitis mouse models (58, 59). The same mouse models showed high serum levels of IL-6, which has a significant role in the balance between Tregs and Th17 cells, after ICI treatment. IL-6 skews transforming growth factor-beta-mediated differentiation of naïve CD4^+^ cells into Tregs towards Th17 differentiation, even by reprogramming Tregs into Th17 cells (60, 61). The serum levels of CXCL1 and IL-6 thus indicate that neutrophil recruitment and the Treg/Th17 balance are important mechanisms in IMC.

In IBD, CXCL1 and IL-6 are secreted by activated macrophages. This cell type may play a significant role in neutrophil recruitment and the skewed Th17 balance in IMC (62, 63). Indeed, in human IMC biopsies macrophages have been reported to upregulate CXCL9/10 expression, alongside their ligand CXCR3 on T-cells (37), and are therefore responsible for recruiting T-cells to a site of Th1-type inflammation (64). CXCR3 deficient mice have shown to be resistant to dextran sulfate sodium-induced colitis (65), highlighting the role of this pathway in the development of colitis. Moreover, macrophage-derived CXCL9 and CXCL10 is also required for T-cell infiltration in tumor sites, indicating the importance of this pathway (66). However, macrophages form a heterogeneous cell population, which has been studied to a limited extent in the context of IMC. Taken together, these data suggest that macrophages potentially have a significant role in T-cell recruitment in IMC. It is therefore to be expected that macrophages are important in more aspects of IMC.

### Anti-Microbial Immunity

The lumen of the colon contains a multitude of mostly bacteria, together referred to as the microbiome. Under certain conditions, some bacteria may become pathogenic. Epithelial tight-junctions, mucus covering the mucosa, and tissue resident macrophages are the first line of defense against such intestinal pathogens. Macrophages detect these pathogens through recognition of exogenous pathogen-associated molecular patterns (67). As a response, macrophages secrete many pro-inflammatory cytokines, such as TNFα, IL-1 and IL-6, but also the anti-inflammatory cytokine IL-10 (68). In ulcerative colitis (UC) and Crohn’s disease (CD), both IBDs, an abnormal reaction to commensal bacteria leads to mucosal inflammation. Several bacteria in IBD stimulate a pathogenic Th1/Th17 response while other bacteria are associated with regulation of Tregs and regulatory B-cells (69). Whether this also applies to IMC is yet to be investigated.

Next to macrophages, Th17 cells are prominent actors in resistance against intestinal pathogens. Interestingly, the composition of commensal bacteria in the gut can skew differentiation of Tregs into Th17 cells (60), a phenomenon that is important in IMC, as discussed above. Noteworthily, a knockout of IL-10R leads to Th17-mediated colitis in regular mice (48), but not in germfree mice (70). This strengthens the idea of a significant role for the microbiome in the onset of UC, and probably also IMC. It is evident that active UC, and most probably also IMC, share a shift toward a Th1/Th17-mediated immune response to the commensal and/or pathogenic microbiota.

Another cell type that leads us to the importance of the microbiome is mucosal-associated invariant T (MAIT) cells. These cells are elevated in gut biopsies of patients with IMC after ipilimumab and nivolumab combination therapy, but not in patients that remained free of adverse events or in patients with UC (71). MAIT-cells are activated indirectly upon bacterial infection and exert antimicrobial properties on bacterial-infected cells (72, 73). The fact that these cells were specifically enhanced in IMC patients, provides a link between the microbiome and IMC that is not seen in similar pathologies. Antimicrobial activity of MAIT-cells against epithelial cells may lead to an impaired barrier function and immune regulation towards intestinal bacteria in patients with IMC.

### Bacterial Strains

The importance of intestinal bacteria has been especially highlighted in mouse models of IMC, induced by oral administration of dextran sulfate sodium prior to anti-CTLA-4 therapy. Treatment with vancomycin, an antibiotic agent that depletes Gram-positive bacteria, reportedly exacerbated severity of IMC histologically and clinically (58, 74). Interestingly, re-introduction of a genus of Gram-positive anaerobic bacteria, *Bifidobacterium* (74) or *Lactobacillus* (58), after vancomycin treatment caused significant amelioration of IMC, both clinically and histologically. Specific strains of these genera, at least *Lactobacillus reuteri, Lactobacillus rhamnosum* and *Bifidobacterium breve*, have shown to be responsible for this positive effect in mice (58, 59).

In humans, Abu-Sbeih and colleagues tested the effect of antibiotic treatment on IMC, including IMD, in a cohort of 826 patients (75). Whereas the use of antibiotics strongly correlated with a lower occurrence of total IMC and IMD, it caused more severe IMC and more hospitalizations. More specifically, anaerobic antibiotics were clinically more detrimental than aerobic antibiotics. This is in accordance with the observations in aforementioned mouse models that Gram-positive anaerobic bacteria were required for IMC resolution (58, 59, 74). The importance of the anaerobic bacterial strains used in those mouse studies is possibly enhanced by it being Gram-positive bacteria that are capable of inducing anti-inflammatory cytokines, rather than induction of only a Th1 secretome by Gram-negative bacteria (76). Nevertheless, the lower overall occurrence of total IMC and IMD following antibiotic therapy in humans, but on the other hand a clinically more severe IMC phenotype, could indicate that IMD and IMC are mechanistically different. Data supporting this hypothesis are currently lacking.

In mouse models of IMC, aiming to get more insight in the underlying bacterial-related mechanisms has yielded various important observations. Anti-CTLA-4 treatment induced a decline in the relative abundance of *Lactobacillus* in stool samples (58). Probiotic *Bifidobacterium* treatment, however, increased the relative abundance of *Lactobacillus*, thereby showing a relation between the two genera (59). These strains may be important to protect the colon against IFNγ-induced epithelial barrier disruption, as shown in human organoid models *in vitro* (40). Any protective function of *Bifidobacterium* is Treg-mediated, since depletion of Tregs abrogated beneficial effects of *Bifidobacterium* in IMC mouse models (59). This bacterial strain caused a genetic upregulation of IL-17R in Tregs of the colonic lamina propria, suggesting Treg behavior in response to IL-17, and thus Th17 cells, may be altered. To date, the effect of IL-17R activation in Tregs remains unknown, but an increase in the receptor for IL-17 might indicate increased sensitivity to Th17 cytokines, allowing Tregs to regulate these cells properly. Tregs may indeed reduce Th17 differentiation and neutrophil infiltration following either *Bifidobacterium* or *Lactobacillus* treatment, since those treatments lead to a decrease in serum levels of IL-6 and keratinocyte-derived chemokine (58, 59).

Another indication for Tregs suppressing inflammation following *Bifidobacterium* administration is the upregulation of the IL-10R on these cells. Interestingly, not only IL-10 was required for attenuation of IMC, but IL-22, a key modulator of epithelial homeostasis (77), also showed to be important (59). This fits with an observation by Wang et al. in mice treated with *Lactobacillus reuteri* (58). They reported that the presence of type 3 innate lymphoid cells (ILC3s), a lymphoid line innate immune cell type known to secrete IL-22 (78), is strongly related to IMC severity. Beneficial probiotic treatment reduced ILC3 cell numbers and improved inflammation in these mice. However, ILC3 cell numbers may be a consequence of IMC, rather than a cause, since crosstalk between ILC3s, macrophages, and the microbiome is reported to be essential for maintaining intestinal homeostasis (79). In addition, a recent study showed that IL-22 producing ILC3s were able to protect against colitis in mice, even when the mice were modified to express abnormal pro-inflammatory secretion profiles (80). However, ILCs, among which those of group 3, are also known for secretion of IL-17 (81, 82), indicating that there could be an ambivalent role for ILCs in IMC.

In general, mouse studies have shed light on the importance of certain genera for protection against IMC. However, fundamental data are limited and thus many other genera or species could be beneficial or detrimental for IMC. Probiotic treatment has not been tested in humans in the context of IMC. Nevertheless, in two out of the three patients who received fecal microbiota transplantation (FMT), a quick reduction of inflammation, as observed by colonoscopy, was noticed (83, 84). Following FMT, *Bifidobacterium* was elevated, even though the patients had a distinct taxonomy from each other prior to FMT (83). This finding might indicate that this particular genus is as important in IMC in humans, as it is in mice.

### Anti-CTLA-4 vs Anti-PD-1

Most studies regarding IMC focus on ipilimumab-induced IMC, either through monotherapy or combination therapy. Several differences in T-cell behavior in IMC between ipilimumab and nivolumab or pembrolizumab treatment are shown (85, 86). In anti-PD-1 treated patients, mucosal infiltration of T-cells was dominated by CD8^+^ T-cells, whereas CD4^+^ dominated after ipilimumab (85). Additionally, ipilimumab led to more epithelial infiltration of lymphocytes and significantly higher levels of mucosal TNFα compared to anti-PD-1 treatment (85, 86). This suggests that mechanisms by which IMC is induced are, to some extent, different between ICI therapies. Furthermore, endoscopic evaluation following anti-PD-1 treatment often does not show aberrations, as opposed to ipilimumab-induced IMC (87). Other than that, mechanistic understanding of IMC and the differences between ICI therapies are mostly suggestive, such as CTLA-4 blockade increasing the numbers of Th17 cells (88), while PD-1 blockade leading to a Th1 dominancy as described in a case report of two IMC patients (89). However, in-depth, head-to-head comparisons are still lacking.

Any additional functional discrepancies between ICI treatments in IMC might be hypothesized by the role of each receptor in colonic homeostasis. In mice, the PD-1/PD-L1 axis is important to maintain tolerance against self-antigens in peripheral tissues, including the gut, by limiting expansion of CD4^+^ and CD8^+^ T-cells (90, 91). That seems to indicate that anti-PD-1 therapy predisposes to intestinal toxicity. However, it has been suggested that PD-L1 can also affect T-cells in the absence of PD-1 (92), thereby possibly remaining functional to some extent after anti-PD-1 blockade. CTLA-4 affects Treg accumulation in the intestinal lamina propria, but not in the thymus, spleen, and mesenteric lymph nodes (93), highlighting its importance in the gut in particular. Considering the difference in frequency of IMC between ICI treatment strategies, CTLA-4 indeed appears to have a more pronounced role in maintaining intestinal homeostasis. The evidence for this difference is mostly suggestive, as data is difficult to compare across studies and different ICI regimens were not studied head-to-head. Hence, it is not yet clear why blockade of CTLA-4 causes IMC more frequently than anti-PD-1 therapy in humans, even though it is clear that both CTLA-4 and PD-1/PD-L1 are important for maintaining mucosal homeostasis in mice.

**Figure 1:**
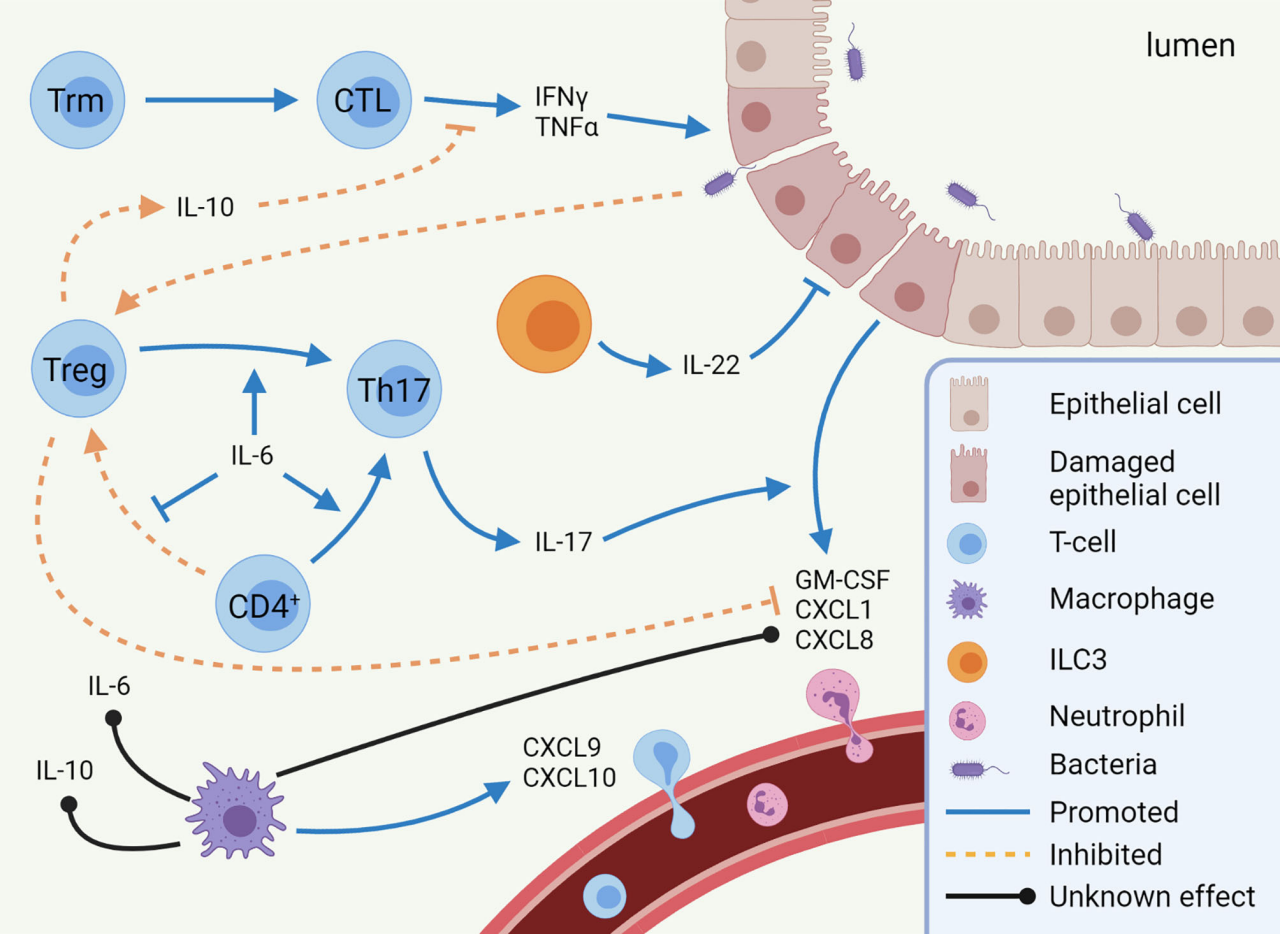
Mechanisms of immune checkpoint inhibitor-mediated colitis (IMC). Pro-inflammatory pathways (CTL, Th17 cells, and neutrophils) are predominantly enhanced in IMC, while anti-inflammatory pathways (Treg differentiation and IL-10 secretion) are inhibited. Other cell types, such as macrophages and ILC3s, are expected to play a role in IMC, but to which extent is unknown. This image was created with BioRender.com. CTL, Cytotoxic T-lymphocyte; CXCL, C-X-C motif chemokine ligand; GM-CSF, Granulocyte-macrophage colony-stimulating factor; IFN, Interferon; IL, Interleukin; ILC, innate lymphoid cell; Th17, T helper 17 cell; TNF, Tumor necrosis factor; Treg, regulatory T-cell; Trm, tissue-resident memory T-cell.

Overall, more evidence is emerging suggesting that some immune cells are predominantly responsible for IMC. As described, in IMC the functional balance between Tregs and Th17s is skewed towards Th17s, leading to increased neutrophil infiltration. Moreover, there is a Th1-dependent inflammatory state, in which in particular IFNγ is suggested to disrupt the epithelial barrier (**Figure 1**). Epithelial permeability leads to interaction between the microbiome and immune cells, although potentially pathogenic microbes and/or commensal microbes that trigger an uncontrolled inflammatory response have not been identified in IMC. However, there are also some subsets for which it is not clear what their exact role is, such as MAIT-cells, ILC3s, and macrophages. In addition, it is not understood why these pathways are induced in some patients and not in others. Answers to these uncertainties may explain the occurrence of immune-related toxicities in certain patients, whereas others remain free of adverse events.

## Biomarkers

In-depth understanding of the mechanisms underlying IMC development is critical to select appropriate immunosuppressive treatments, or to prevent the development of IMC. Another way to reduce the incidence and severity of IMC is to identify markers which predict patients at risk of developing IMC, either all grade or specifically high-grade toxicity. Being able to predict the risk of IMC for patients allows closer monitoring of those that are likely to develop high-grade toxicities, or enables selection of an alternative anti-cancer treatment.

### Cellular Indicators

Several cell types are involved in or correlate with IMC. Cellular products or even the mere presence of cells are potential candidates for biomarkers of IMC development.

As already discussed, IL-17 secreting Th17s are important mediators. While baseline IL-17 serum levels do not correlate with all grade ipilimumab-induced IMC occurrence (50), it significantly correlated with grade 3 IMC in a cohort of 33 patients (94). Baseline serum IL-17 is therefore a potential marker for high-grade colitis, although it remains to be confirmed in larger cohorts.

Another cell type that is abundantly present in IMC is neutrophils. A high neutrophil to lymphocyte ratio (NLR) in serum is known to correlate with worsened ICI clinical outcome (95–97). Although its predictive correlation with all irAEs is mostly weak, NLR distinguishes grade 3 and higher irAEs from low grade irAEs after pembrolizumab therapy and it can be used to monitor the onset of irAEs (98, 99). For IMC in particular, a baseline NLR higher than 5 correlated with development of IMC (100). However, in the same study, a validation cohort failed to show a significant correlation between NLR and IMC. Another interesting marker related to neutrophils is the genetic expression of *CD177*, a modulator of neutrophil migration (101), in circulating cells. At week 3 after the first ipilimumab treatment, this marker showed high specificity for predicting patients who later developed gastrointestinal adverse events (102). However, the sensitivity was low in this study, meaning *CD177* is unable to capture all patients at risk of IMC on its own.

Other potential neutrophil-related biomarkers are based on similarities with IBD. Fecal calprotectin and lactoferrin are established markers for active inflammation in IBD (103). Calprotectin is abundantly present in the cytoplasm of phagocytes and has pro-inflammatory functions upon secretion (104). A major source of calprotectin release is cell death of neutrophils (105). Lactoferrin is, amongst others, released in granules by activated neutrophils (106). Neutrophil infiltration is often observed in IMC biopsies. Accordingly, levels of fecal calprotectin and lactoferrin correlate with endoscopic findings of ulceration and histological signs of IMC (107). Furthermore, fecal calprotectin is increased upon the onset of diarrhea and reduced when clinical remission is observed (108, 109). This could therefore be a promising marker to monitor disease activity and relapse in patients, as already suggested in American Society of Clinical Oncology guidelines (110). The predictive value of fecal calprotectin and lactoferrin has not yet been investigated. However, since these are both markers for neutrophil infiltration, distinguishing IMC from an IBD exacerbation will not be possible for IBD patients who underwent ICI therapy (111, 112).

### Microbiota

At a bacterial level, some potential biomarkers have been reported. In two patient cohorts of 34 and 55 patients, microbiota composition analysis was performed on feces of patients prior to the start of ICI therapy for metastatic melanoma. In feces of patients later developing IMC, several families of the *Bacteroidetes* phylum were underrepresented (113, 114). The same observation was made for IMD in a cohort of 26 patients with lung cancer, which may suggest a gut protective role of this phylum (115). The *Firmicutes* phylum, on the other hand, was increased at baseline for patients later developing IMC (114, 115). Thus, a high ratio of *Firmicutes* to *Bacteroidetes* at baseline measurements of feces may provide predictive insight in which patients are likely to develop IMC, although these observations should be validated in larger patient cohorts to test clinical applicability. Whereas IMC has overlapping characteristics with several IBDs, a low *Firmicutes* to *Bacteroidetes* ratio is actually seen in CD (116). This indicates a different role of these bacterial families in IMC and CD.

Looking at resistance to IMC development rather than risk of development, polyamine transport units in bacteria may be beneficial. A prediction model using molecular levels of these polyamine transport units showed a sensitivity of 70% and a specificity of 100% for resistance to IMC development, indicating all patients that were predicted to develop IMC indeed did so, however, 30% of patients were false negatively assigned to remain free of IMC (113). Interestingly, blocking polyamine reduces the number of tumor-infiltrating immune suppressor cells, such as myeloid-derived suppressor cells, Tregs and M2 macrophages, thereby boosting the antitumor response in mouse models (117, 118). Hence, the microbiome might exert a suppressive function in the immune response through polyamine transport, which could explain its correlation with resistance to IMC.

### Other Markers

While most of the potential biomarkers reported so far focused on neutrophils, Th17 cells, or the microbiome, there are also some markers that are less specific. In IBD, vitamin D intake has been reported to improve clinical outcomes (119). The importance of vitamin D is underscored in mice: immune cells from vitamin D deprived mice do show increased IL-17 and IFNγ secretion, failure to develop essential anti-inflammatory T-cell subsets, and disruption of the epithelial barrier, all of which are important mechanisms of IMC (120, 121). Indeed, vitamin D intake during ICI treatment was found to be strongly correlated with reduced risk of IMC development in a cohort of 213 patients, which was additionally validated on an independent cohort of 169 patients (100). Although this does not necessarily mean that vitamin D has a predictive value in this context, it is interesting to take vitamin D into account in the clinic, particularly in case of an insufficiency.

For irAEs in general, a wide range of predictive markers is studied. For instance, a large multi-omics study showed that a bivariate model using ADPGK and LCP1, which are both related to T-cell activation, is a promising prediction tool (122). Since such markers are not specific for IMC, we would like to refer the reader to some reviews on this topic (123, 124). While some of these markers provide a decent predictive value, it is mostly unclear whether these are applicable for IMC specifically. Such general markers, however, are definitely of interest to investigate in prospective studies regarding IMC.

## Mechanism-Based Future Research and Approaches to Management

It is well established that Th17 cells, derived from Tregs or naïve T-cells, are important actors in IMC. Also, CTLs are thought to be pathogenic in IMC by disrupting the epithelial barrier and creating a state of inflammation. However, many questions still remain. It is often unclear which signals induce these cell developments, or why this signaling is evoked in certain patients. Is it directly or indirectly related to ICI therapy? In other words, does ICI treatment lead to attraction of macrophages and skewing towards Th17 cells, or is it secondary to e.g., activation of autoreactive B or T-cells? Moreover, there is still a lot to be elucidated about tissue-resident T-cells. For instance, CTLs appear to be to be partly derived from Trms, although its mechanism is unknown. In addition, several resident T-cell types involved in interactions with the microbiome, ILC3s, MAIT-cells, and macrophages, are indicated to be affected. While macrophages are suggested to promote T-cell recruitment, it is likely that their role in IMC is larger. Their secretome has strong overlap with several cytokines and chemokines that are expressed in IMC. Yet, many studies have focused on the role of T-cells in IMC. ILC3s and MAIT-cells may have more protective, antimicrobial roles. Knowledge on how these cell types are behaving in IMC is important for understanding the role of potentially pathogenic bacteria.

To answer these remaining questions, future research should focus on specific mechanisms of IMC development. Cellular composition and involved cytokines and chemokines in baseline and on-treatment sigmoid biopsies should be compared in ICI-treated patients who developed IMC. With the use of several advanced techniques, such as RNA-sequencing, multiplex immunohistochemistry, and flow cytometry, cellular and molecular data can be readily harvested from these biopsies. The microbiome should also be taken into account in prospective studies, considering its significant role. Especially those microbes in close contact with the mucosal tissue should be examined and differences in host-microbe interactions in the mucosa of patients with IMC versus patients remaining free of IMC should be explored. In future IMC-focused trials, blood, colon biopsies, and stool should be collected at standardized points in time, e.g., at baseline and during ICI cycles. Understanding the interactions between all key players in IMC is of utmost importance to improve the current clinical treatments. This research may lead to additional targets for treatment, as well as biomarkers that could identify patients at risk of high-grade IMC.

Currently, several guidelines suggest that patients diagnosed with high-grade IMC are to be treated with first-line systemic corticosteroids (110, 125, 126). In case of steroid-refractory IMC, anti-TNFα treatment with infliximab is often initiated. However, both treatments are unspecific for IMC and therefore come with several drawbacks, such as risk of infection and drug-induced comorbidities (127, 128). Infliximab has even been observed to compromise the long-term anti-tumor response in steroid refractory patients (129).

Recently, the use of immunosuppressants targeting specifically the gut in IMC has been investigated, primarily vedolizumab. This antibody blocks the α4β7 integrin, which is involved in homing of T-cells to the gut (130). Vedolizumab has adequately replaced infliximab in steroid-refractory patients, and administration within 10 days of IMC onset leads to better management and clinical remission (131, 132). However, histologic remission is often not seen six months after clinical remission, indicating that there is room for improvement (131). Prospective studies interfering with alternative pathways may provide more options for IMC-specific treatments.

**Figure 2:**
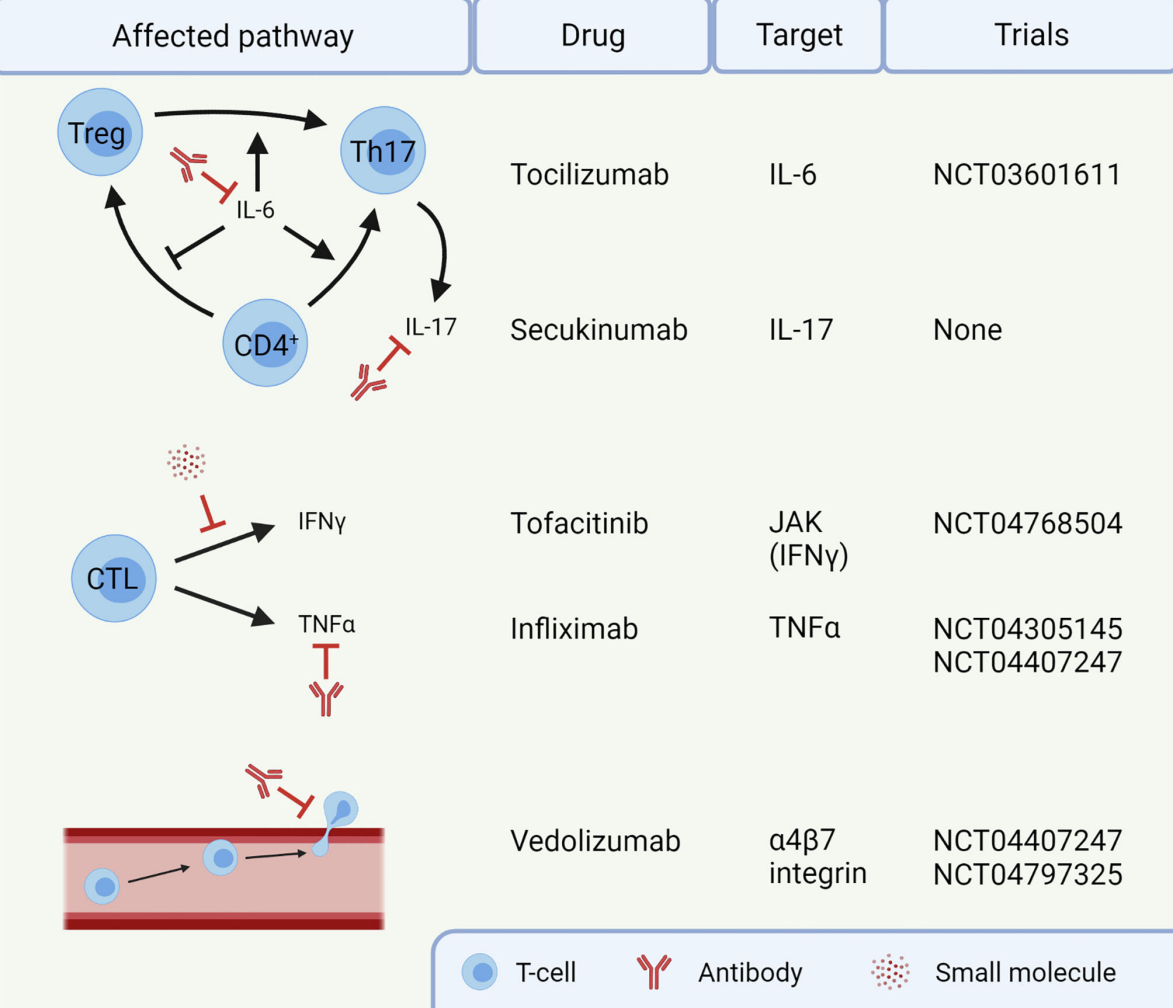
Targets for treatment of immune checkpoint inhibitor-mediated colitis (IMC). Infliximab and vedolizumab are already standard of care in steroid-refractory IMC. The other agents are currently not routinely given to patients. This image was created with BioRender.com. CTL, Cytotoxic T-lymphocyte; IFN, Interferon; IL, Interleukin; JAK, Janus kinase; Th17, T helper 17 cell; TNF, Tumor necrosis factor; Treg, regulatory T-cell.

Several potential targets for IMC are already in clinical trials (**Figure 2**). For instance, blocking IL-6 with tocilizumab could reduce Th17 differentiation, thereby restoring the dysfunctional balance between Tregs and Th17 cells (NCT03601611). Additionally, cytokine secretion by Th17 cells could be targeted using secukinumab, an anti-IL-17A monoclonal antibody. Secukinumab has already shown a beneficial therapeutic effect in patients suffering from ICI-induced psoriasis, without affecting their anti-tumor response (133). Caution is required when using this antibody to treat IMC, since secukinumab is ineffective in CD, risking fungal infections along the way (134). In UC, an antagonist of the p40 subunit of IL-12 and IL-23, called ustekinumab, showed to induce and maintain disease remission (135). It has not been studied in the context of IMC and the cytokines IL-12 and IL-23 have not been reported to be important in IMC yet. IFNγ, on the other hand, does have an important role in IMC, causing a pro-inflammatory response and epithelial damage. The function of IFNγ can be inhibited by targeting the JAK signaling pathway with tofacitinib. Tofacitinib has shown efficacy against IMC in five patients (136, 137) and will be investigated in a clinical trial with ten patients (NCT04768504). Tofacitinib has also shown efficacy in treatment of IBD (138). However, JAK signaling is reported to be important for an anti-tumor response upon ICI therapy (139), so caution with inhibition of this pathway in IMC is necessary. Future IMC trials should focus on mechanism-based approaches for selection of first-line immunomodulating agents. Such agents should interfere with IMC, without compromising the efficacy of ICI antibodies.

In addition to interfering with pathways of the immune system, targeting the microbiome is also an option for treatment of IMC. For instance, an experimental FMT immediately showed alleviation of IMC symptoms in patients refractory to corticosteroids, infliximab, and vedolizumab (83, 84). FMT has already shown promising therapeutic effects in *Clostridoides difficile* infections (140). Recently, a large clinical trial, 800 patients with any stage melanoma, non-small cell lung cancer or genitourinary cancer, has been set up to study potential biomarkers in the microbiome and the safety and efficacy of FMT in IMC (NCT03819296). An alternative to FMT would be the use of probiotics. Probiotics are effective in mouse models of IMC, and successfully used against necrotizing enterocolitis in human preterm infants (141). Since FMT and probiotics aim to normalize the gut microbiome, it is an attractive strategy to treat IMC without affecting the efficacy of ICI therapy. The composition of the gut microbiome can affect the antitumor response negatively or positively (142, 143). Promising is the observation in mouse models that probiotic treatment with two different bacterial genera attenuates IMC without compromising the antitumor response (58, 74). Therefore, IMC treatment with specific bacterial strains might be more suitable than unspecific FMT treatment with the risk of lowering the anticancer activity of the immune system.

All in all, it is expected that ICI therapy becomes available for more types of cancer in upcoming years (144, 145). To reduce physical harm and loss of quality of life due to irAEs, the balance between efficacy and toxicity requires optimization. Results of mechanism-based IMC research may lead to optimization of treatments and predictions of IMC. In addition, it may provide new insights concerning non-intestinal irAEs. We envision direct clinical relevance for future patients undergoing ICI therapy, in which severe irAEs with quality-of-life deterioration can be treated or even be prevented.

## Author Contributions

HW and IV conceptualized this review. HW, MS, and MG were responsible for writing the manuscript. All authors contributed to the article and approved the submitted version.

## Funding

This work was supported by NWO-Vici grant (918.14.655) to IV and EU grant 825410 (Oncobiome).

## Conflict of Interest

FH has served on advisory boards, as speaker, or consultant for AbbVie, Celgene, Janssen-Cilag, Merck Sharp & Dohme, Takeda, Celltrion, Teva, Sandoz, and Dr Falk, and has received unrestricted grants from Dr Falk, Janssen-Cilag, and AbbVie. MH received research grants from Merck and AstraZeneca. BP received fees from advisory boards of Takeda, Bristol-Myers Squibb, Janssen, and Pfizer. BP received lecturing fees from AstraZeneca and Pfizer.

The remaining authors declare that the research was conducted in the absence of any commercial or financial relationships that could be construed as a potential conflict of interest.

## Publisher’s Note

All claims expressed in this article are solely those of the authors and do not necessarily represent those of their affiliated organizations, or those of the publisher, the editors and the reviewers. Any product that may be evaluated in this article, or claim that may be made by its manufacturer, is not guaranteed or endorsed by the publisher.
